# Socioeconomic disparities in the trends of inpatient utilization for middle-aged and older adults in China: the perspective of international comparison from four cohorts

**DOI:** 10.1186/s41256-025-00464-4

**Published:** 2025-12-19

**Authors:** Yemin Yuan, Huaxin Si, Yiqi Xia, Yanshang Wang, Zhenyu Shi, Ping He

**Affiliations:** 1 School of Public Health, Peking University, Beijing, 100191 China; 2 China Center for Health Development Studies, Peking University, 38 Xue Yuan Road, Haidian District, Beijing, 100191 China; 3 National Health Commission Key Laboratory of Health System Reform and Governance, Peking University, Beijing, 100191 China; 4 School of Health and Life Sciences, University of Health and Rehabilitation Sciences, No. 369, Qingdao National High-Tech Industrial Development Zone, Qingdao, 266071 China; 5 Beijing Institute for Health Development, Peking University, Beijing, 100191 China

**Keywords:** Healthcare utilization, Trends, Inpatient utilization, Socioeconomic status

## Abstract

**Background:**

During the past decade, China has witnessed a rapid increase in healthcare utilization. However, whether this surge in healthcare use is reasonable remains an urgent question to be answered, particularly for informing scientific policy design and adjustment in future health systems reform**.** We aimed to analyze the differences in healthcare utilization trends among Chinese adults aged 50 and above in comparison with other countries and regions, and to investigate the association between socioeconomic status (SES) and healthcare utilization in China.

**Methods:**

Participants aged 50 and over were drawn from the ageing surveys conducted in China, the United States, South Korea, and Europe. SES was measured using education level, total household income, and employment status. Random-effects negative binomial regression models were employed to examine the association between SES and healthcare utilization.

**Results:**

From 2011 to 2020, the outpatient utilization rates in China showed no clear long-term upward or downward trend. The inpatient utilization rates in the United States (*tau-b* = − 0.02, *P* < 0.001) and South Korea (*tau-b* = − 0.05, *P* < 0.001) declined gradually, whereas inpatient utilization rates in China continuously grew (*tau-b* = 0.08, *P* < 0.001). In China, inpatient utilization rates across different SES groups generally showed an upward trend (*P* < 0.01). Elementary and lower education was positively associated with inpatient utilization rates compared to middle school and higher education (IRR = 1.11, 95% CI: 1.04–1.18, *P* = 0.003). Similar patterns were observed for low household income (IRR = 1.08, 95% CI: 1.02–1.14, *P* = 0.007), non-employment (IRR = 1.39, 95% CI: 1.32–1.47, *P* < 0.001) and retirement (IRR = 1.38, 95% CI: 1.26–1.52, *P* < 0.001). These associations remained significant even among individuals covered by the Urban and Rural Resident Basic Medical Insurance (URRBMI). Participants with lower SES were more likely to report having two or more chronic diseases and poor self-rated health (*P* < 0.001).

**Conclusions:**

Inpatient utilization rates among middle-aged and older adults in China have experienced excessive growth over the past decade. Low SES was associated with high inpatient utilization, likely attributable to the poorer health status of low-SES groups. This association persisted in people covered by URRBMI. Policy reforms should prioritize the development of primary care, targeted health management, and equitable adjustments to health insurance. These measures are essential for curbing unnecessary hospitalizations and advancing healthcare equity in China.

**Supplementary Information:**

The online version contains supplementary material available at 10.1186/s41256-025-00464-4.

## Background

Ageing populations, epidemiological transitions toward chronic diseases, and evolving health policy have reshaped global health service demands [1, 2]. Trends in healthcare utilization exhibit significant variation across countries and regions, influenced by factors such as population demographics, economic development, and healthcare policy [3]. These trends play a pivotal role in shaping health policy, as they reflect the healthcare needs and the ability of the population to access and use available services. Despite progress in universal health coverage (UHC) initiatives, the Organization of Economic Co-operation and Development (OECD) highlights widening disparities in healthcare access between high- and low-income countries [4]. As the world’s most populous nation, healthcare utilization trends in China hold profound implications for global health governance and sustainable development goals (SDGs) attainment.

China has experienced remarkable growth in healthcare utilization over the past decade. The total number of medical visits at healthcare institutions nationwide increased from 6.89 billion in 2012 to 8.47 billion in 2021, while inpatient admissions rose from 178.12 million in 2012 to 247.26 million in 2021 [5, 6]. By 2023, the total number of medical visits reached 9.55 billion, with an average of 6.8 medical visits per capita annually, and inpatient admissions numbered 301.87 million, reflecting an annual hospitalization rate of 21.4% among the population [7]. However, the rationality of this surge in healthcare service utilization and its comparison with high-income countries remain unclear, particularly amid China’s stark regional and urban–rural disparities in medical resources [8, 9].

Since the launch of China’s new round of health systems reform in 2009, the Chinese government has aimed to provide equitable and affordable health care for all the people [10, 11]. The Urban Employee Basic Medical Insurance (UEBMI) and the Urban and Rural Resident Basic Medical Insurance (URRBMI) constitute the current basic social health insurance programs in China [10, 12, 13]. UEBMI covers urban formal-sector workers with higher funding (employer-employee co-contributions) and average reimbursement rates (~ 70% for inpatient care); and URRBMI covers rural residents and urban non-employees with lower funding (government subsidies plus individual premiums) and lower inpatient reimbursement rates (~ 50–60%) [10]. The expansion of health insurance coverage has achieved remarkable progress since 2009, reaching over 95% of the Chinese population by 2013 and remaining stable thereafter [11, 14]. However, disparities in healthcare utilization persist a critical challenge in China [11, 12].

Socioeconomic status (SES) is a key determinant of healthcare utilization [15]. Extensive evidence exists from high-income countries [16, 17] and some low- and middle-income countries [18, 19]. Existing studies primarily focused on specific populations, such as children [20], patients undergoing certain surgeries [21–23], or those suffering from specific types of diseases [24–27]. Low SES populations often face higher burdens of chronic diseases and poorer baseline health [9, 28], which increases the need for healthcare. Additionally, they encounter greater barriers to primary care access [29], leading to delayed treatment of mild conditions that progress to severe stages requiring hospitalization. Lower health literacy among low SES groups also contributes to suboptimal healthcare and timely symptom management [30].

Due to differences in the health systems and sociodemographic characteristics, the socioeconomic patterns of healthcare utilization in China often differ from those observed in other countries. The Chinese healthcare system operates through a three-tiered service delivery model consisting of primary care facilities (community health centers), secondary hospitals (county/district-level), and tertiary hospitals (provincial/municipal-level) [31]. However, this hierarchy faces practical challenges: the quality and capacity of primary care institutions remain uneven, with limited access to advanced diagnostics and skilled providers in many regions [32]. Concurrently, the referral system—designed to guide patients from primary care to higher-tier hospitals—operates imperfectly in practice, as weak coordination between institutions and low public trust in primary care often led patients to bypass primary care facilities and directly seek care at higher-tier hospitals [33]. Currently, research on the association between socioeconomic factors and healthcare utilization in China remains limited.

In this study, we aimed to address two critical knowledge gaps. First, using the international cohorts of ageing, we compared the differences in the trends of healthcare utilization among adults aged 50 and above in China with other countries and regions. Second, we further investigated the association between SES and healthcare utilization in China, considering contextual factors like health insurance type.

## Methods

### Study design and data sources

In our international comparative analysis, we used data from the China Health and Retirement Longitudinal Study (CHARLS) [34], the Health and Retirement Study (HRS) [35], the Korean Longitudinal Study of Aging (KLoSA) [36], and the Survey of Health, Ageing and Retirement in Europe (SHARE) [37]. In this study, we used data from a similar time range: 2011–2020 for CHARLS, 2012–2020 for HRS, 2012–2020 for KLoSA, 2011–2019 for SHARE. We excluded participants who were younger than 50 years of age. Participants were also excluded if they had missing information on healthcare utilization and sociodemographic variables (age, sex, residence and marital status). Flowcharts of sample selection are shown in Supplementary Figure S1. Finally, 75,368 observations from CHARLS, 78,042 observations from HRS, 35,341 observations from KLoSA, and 233,093 observations from SHARE were available for the analysis. See Table S1 for details. In a subsequent CHARLS analysis, we further excluded participants who had missing values of socioeconomic status or covariates, resulting in 44,137 observations (Figure S2).

### Measurements

In this study, we used education, total household income, and employment status to measure socioeconomic status (SES). Education was classified as elementary school and below or middle school and above. The total household income was divided into low and high levels according to the median. Employment status was classified as not working, working, or retired. Health insurance was classified as no insurance (none), UEBMI, URRBMI, or others. Other insurance schemes include government medical insurance, medical aid, private medical insurance, urban non-employed person’s health insurance, long-term care insurance, and other medical insurance.

Healthcare utilization included outpatient visits and inpatient admissions. We defined outpatient utilization rate as the annual number of visits per capita and defined inpatient utilization rate as the annual number of admissions per capita. In CHARLS, the time span of outpatient utilization was the past month, and the time span of inpatient utilization was the past year. In HRS and KLoSA, the time span for both outpatient and inpatient utilization and costs was the last two years. In SHARE, the time span of outpatient and inpatient utilization was the past year (Table S2). For a better understanding, all outcome variables were uniformly converted to one-year time units [38].

Other covariates included age (50–59, ≥ 60 years), sex (male, female), residence (urban, rural), marital status (married or partnered, unmarried and others covered separated, divorced, and widowed), smoking (no, yes), alcohol drinking (no, yes), number of chronic diseases (0, 1, ≥ 2), self-reported health status (good, fair, and poor), and survey years (2011, 2013, 2015, 2018, 2020).

### Statistical analysis

We used the Mann–Kendall trend test to examine the time trends in the annual number of outpatient visits and inpatient admissions per capita in CHARLS, HRS, KLoSA and SHARE. In CHARLS, we also used the Mann–Kendall trend test for the trend of inpatient admissions among different SES populations. In the same survey year, the Wilcoxon rank-sum test was used for comparison between two groups and the Kruskal–Wallis H test was used for comparison between multiple groups. All descriptive analyses were weighted to account for the complex, multistage design of the study, and non-response in multinational data.

We used random-effects negative binomial regression models to investigate the association between SES and inpatient utilization because the variances were greater than the means in outcome variables (mean = 0.26, variance = 0.81). This indicates that excessive overdispersion was present in the outcome variables (likelihood ratio test of *P* < 0.001). The negative binomial model allows for overdispersion by assuming that the individual error terms come from a particular probability distribution (the γ distribution) [39]. Additionally, we performed a likelihood ratio test comparing the random-effects negative binomial model with a pooled negative binomial model (without individual-level random effects). The results revealed inter-individual variability, validating the necessity of incorporating random effects. The command in Stata is “xtnbreg”. We reported incidence rate ratio (IRR) and 95% confidence interval (CI). We utilized four models in the analyses, of which Model 1 was a crude model. In Model 2, we accounted for survey years, age, sex, residence, and marital status. Model 3 was further included smoking and alcohol drinking based on Model 2. Model 4 was fully adjusted to include all covariates. We did a sensitivity analysis. We investigated the association between SES and inpatient utilization using Poisson models instead of negative binomial models. The command in Stata is “xtpoisson”.

To explore the differential effect in population groups, we did subgroup analyses, stratified by membership of insurance schemes, using the same regression analyses but with the stratification variable removed. All analyses were performed by Stata (version 15.0). We considered *P* values of less than 0.05 to be significant.

## Results

### Trends in healthcare utilization in four international cohorts of ageing

**Fig. 1:**
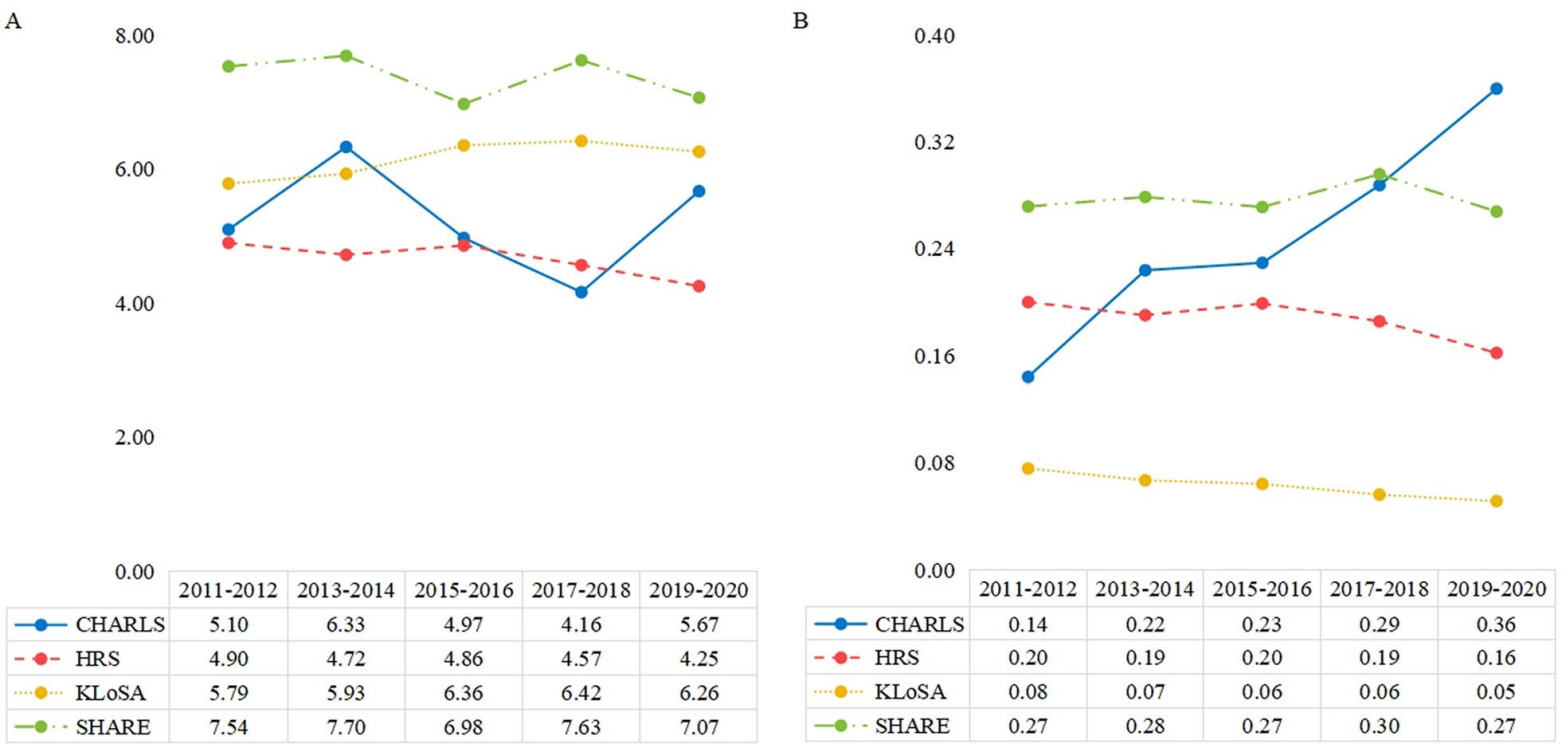
The annual number of outpatient visits and inpatient admissions per capita (**A** outpatient visits; **B** inpatient admissions). Notes: *CHARLS* China Health and Retirement Longitudinal Study; *HRS* Health and retirement study; *KLoSA* Korean Longitudinal Study of Aging; *SHARE* Survey of Health, Ageing and Retirement in Europe

Figure 1 and Table S3 present the outpatient and inpatient utilization rates across the four international cohorts of ageing. The outpatient utilization rates in CHARLS fluctuated over the years, showing no clear long-term upward or downward trend (*tau-b* = 0.00, *P* = 0.269). The outpatient utilization rates in HRS gradually decreased over the past decade at a modest pace (*tau-b* = − 0.02, *P* < 0.001), while those in KLoSA showed a steady upward trend (*tau-b* = 0.02, *P* < 0.001).

Similarly, the inpatient utilization rates varied across cohorts. In HRS and KLoSA, the inpatient utilization rates declined gradually from 2012 to 2020 (HRS: *tau-b* = − 0.02, *P* < 0.001; KLoSA: *tau-b* = − 0.05, *P* < 0.001). However, in CHARLS, the inpatient utilization rates consistently increased from 2011 to 2020 (*tau-b* = 0.08, *P* < 0.001). By 2020, the inpatient utilization rates in CHARLS were significantly higher than those in the other three cohorts.

### Sample Characteristics in CHARLS

Table 1 summarizes the sample characteristics of CHARLS. A total of 44,137 observations were eligible for inclusion in our analysis. The majority of the sample, 27,653 (62.7%), were aged 60 years or older. 22,311 (50.5%) were female. Educational attainment was relatively low, with 30,123 (68.2%) having primary education or below. 21,495 (48.7%) were categorized as high-income households. Employment status displayed that 25,177 (57.0%) were working, and 5,555 (12.6%) were retired. Health insurance coverage was nearly universal, with 42,208 (95.6%) having at least one type of health insurance. The detailed characteristics of the CHARLS sample, stratified by survey year, are shown in Table S4.

**Table 1:** Sample characteristics in CHARLS

Characteristics	Total (n = 44,137)
Socioeconomic status	
Education, n (%)	
Elementary school and below	30,123 (68.2)
Middle school and above	14,014 (31.8)
Total household income, n (%)	
Low income	22,642 (51.3)
High income	21,495 (48.7)
Employment status, n (%)	
Not working	13,405 (30.4)
Working	25,177 (57.0)
Retired	5,555 (12.6)
Health insurance, n (%)	
None	1,929 (4.4)
UEBMI	5,013 (11.4)
URRBMI	36,072 (81.7)
Others^1^	1,123 ( 2.5)
Other covariates	
Age (years), n (%)	
50–59	16,484 (37.3)
≥ 60	27,653 (62.7)
Sex, n (%)	
Male	21,826 (49.5)
Female	22,311 (50.5)
Residence, n (%)	
Urban	15,833 (35.9)
Rural	28,304 (64.1)
Marital status, n (%)	
Married or partnered	7,178 (16.3)
Unmarried and others^2^	36,959 (83.7)
Smoking, n (%)	19,445 (44.1)
Alcohol drinking, n (%)	19,565 (44.3)
Number of chronic diseases, n (%)	
0	9,665 (21.9)
1	11,075 (25.1)
≥ 2	23,397 (53.0)
Self-reported health status, n (%)	
Good	16,962 (38.4)
Fair	18,074 (40.9)
Poor	9,101 (20.6)
Survey years	
2011	6,180 (14.0)
2013	7,914 (17.9)
2015	6,138 (13.9)
2018	10,622 (24.1)
2020	13,283 (30.1)

*CHARLS* China Health and Retirement Longitudinal Study; *UEBMI* Urban Employee Basic Medical Insurance; *URRBMI* Urban and Rural Resident Basic Medical Insurance

^1^Others include government medical insurance, medical aid, private medical insurance, urban non-employed person’s health insurance, long-term care insurance, and other medical insurance

^2^Others covered separated, divorced, and widowed

### Trends in inpatient utilization in CHARLS by SES

**Fig. 2:**
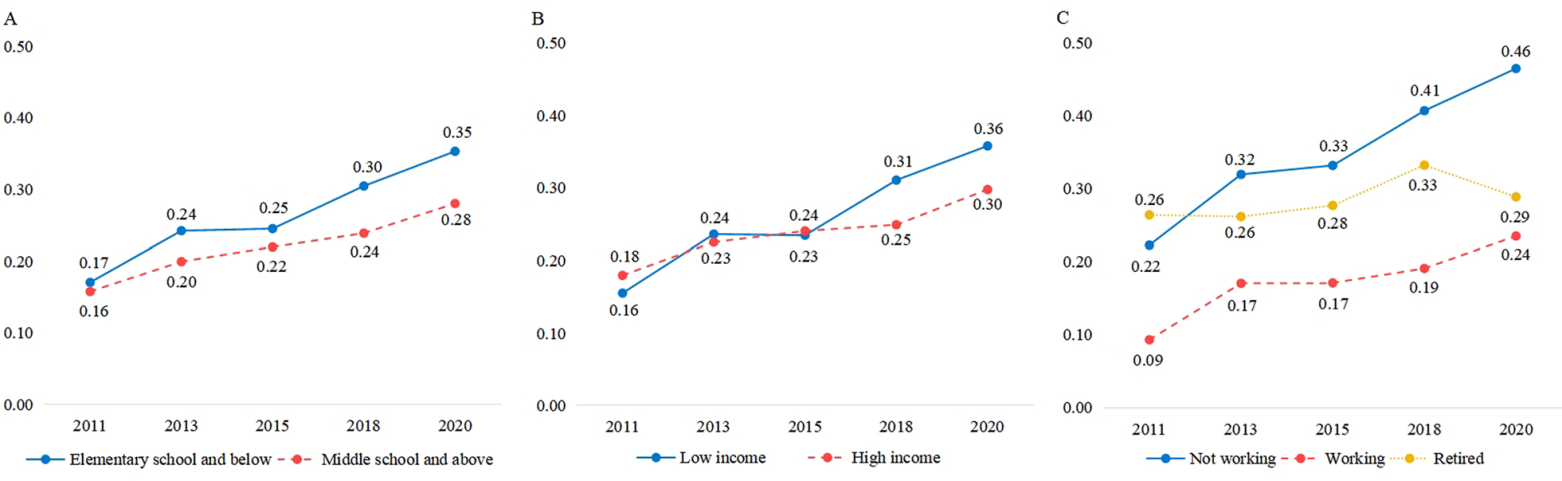
The annual number of inpatient admissions per capita by socioeconomic status (**A** education; **B** total household income; **C** employment status)

The inpatient utilization rates across different SES groups exhibited a general upward trend over time (*P* < 0.01), as illustrated in Fig. 2 and detailed in Table S5. In 2013, 2018 and 2020, participants with elementary school and below education had significantly higher inpatient utilization rates compared to those with middle school and higher education (2013: *z* = 2.10, *P* = 0.036; 2018: *z* = 3.83, *P* < 0.001; 2020: *z* = 4.26, *P* < 0.001). Additionally, household income levels exhibited different trends: in 2011, participants with high household income had higher inpatient utilization rates than those with low income (*z* = -1.99, *P* = 0.046), but this trend reversed in 2018 (*z* = 3.82, *P* < 0.001) and 2020 (*z* = 3.87, *P* < 0.001), with low-income participants exhibiting higher utilization rates. There were statistically significant differences in inpatient utilization rates among participants with different employment status (*P* < 0.01).

### Association between SES and inpatient utilization among middle-aged and older adults in China

Table 2 shows the association between SES and inpatient utilization. In the crude model (Model 1), elementary and lower education was positively associated with inpatient utilization compared to middle school and higher education (IRR = 1.13, 95% CI: 1.06–1.20, *P* < 0.001). Similar trends were observed for low household income (IRR = 1.11, 95% CI: 1.06–1.17, *P* < 0.001), non-employment (IRR = 1.79, 95% CI: 1.70–1.89, *P* < 0.001) and retirement (IRR = 1.56, 95% CI: 1.44–1.70, *P* < 0.001). These associations remained statistically significant but were slightly attenuated in the fully adjusted model (Model 4). The results of the Poisson regression are consistent (Table S6). The association between SES and inpatient utilization across different survey years is further detailed in Table S7.

**Table 2:** Association between socioeconomic status and inpatient utilization among middle-aged and older adults in China

	Model 1		Model 2		Model 3		Model 4	
	IRR (95% CI)	*P* value	IRR (95% CI)	*P* value	IRR (95% CI)	*P* value	IRR (95% CI)	*P* value
Education (ref: middle school and above)								
Elementary school and below	1.13 (1.06–1.20)	< 0.001	1.18 (1.10–1.26)	< 0.001	1.17 (1.09–1.25)	< 0.001	1.11 (1.04–1.18)	0.003
Total household income (ref: high income)								
Low income	1.11 (1.06–1.17)	< 0.001	1.16 (1.09–1.22)	< 0.001	1.15 (1.09–1.22)	< 0.001	1.08 (1.02–1.14)	0.007
Employment status (ref: working)								
Not working	1.79 (1.70–1.89)	< 0.001	1.69 (1.59–1.79)	< 0.001	1.67 (1.57–1.77)	< 0.001	1.39 (1.32–1.47)	< 0.001
Retired	1.56 (1.44–1.70)	< 0.001	1.56 (1.42–1.72)	< 0.001	1.56 (1.41–1.71)	< 0.001	1.38 (1.26–1.52)	< 0.001

*IRR* incidence rate ratio; *CI* confidence interval

Model 1 was crude model. Model 2 was adjusted for survey years, age, sex, residence, marital status, and health insurance. based on Model 1. Model 3 was adjusted for smoking and alcohol drinking based on Model 2. Model 4 was adjusted for number of chronic diseases and self-reported health status based on Model 3

**Fig. 3:**
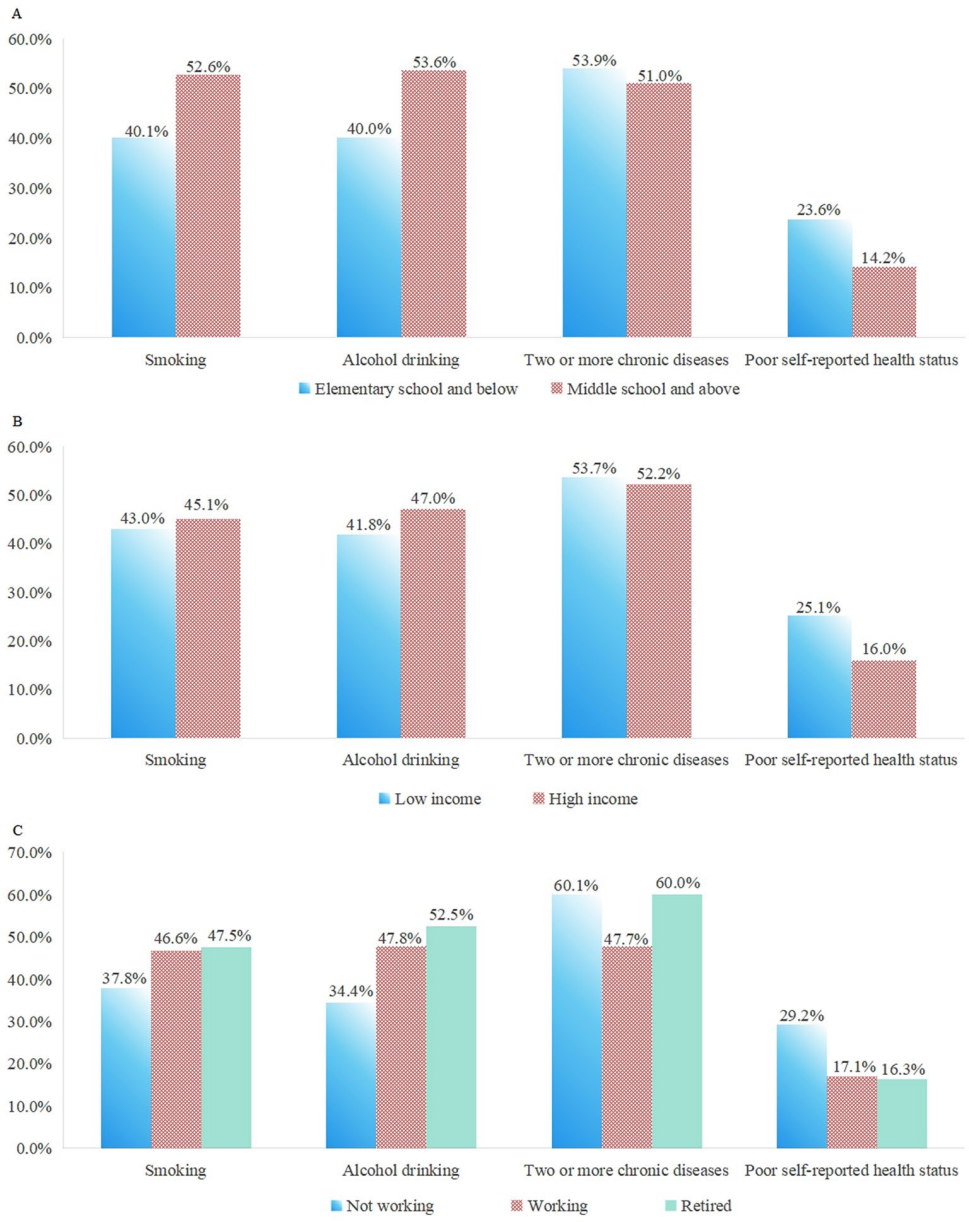
The proportion of health behaviors and health status among people of different socioeconomic status (**A** education; **B** total household income; **C** employment status)

Figure 3 and Table S8 illustrate the proportions of health behaviors and health status across different SES groups. Participants with low SES had higher proportions of chronic disease burden, with a greater likelihood of suffering from two or more chronic diseases, and were more likely to report poor self-rated health compared to those in higher SES groups (*P* < 0.001).

### Subgroup analysis by health insurance coverage

**Fig. 4:**
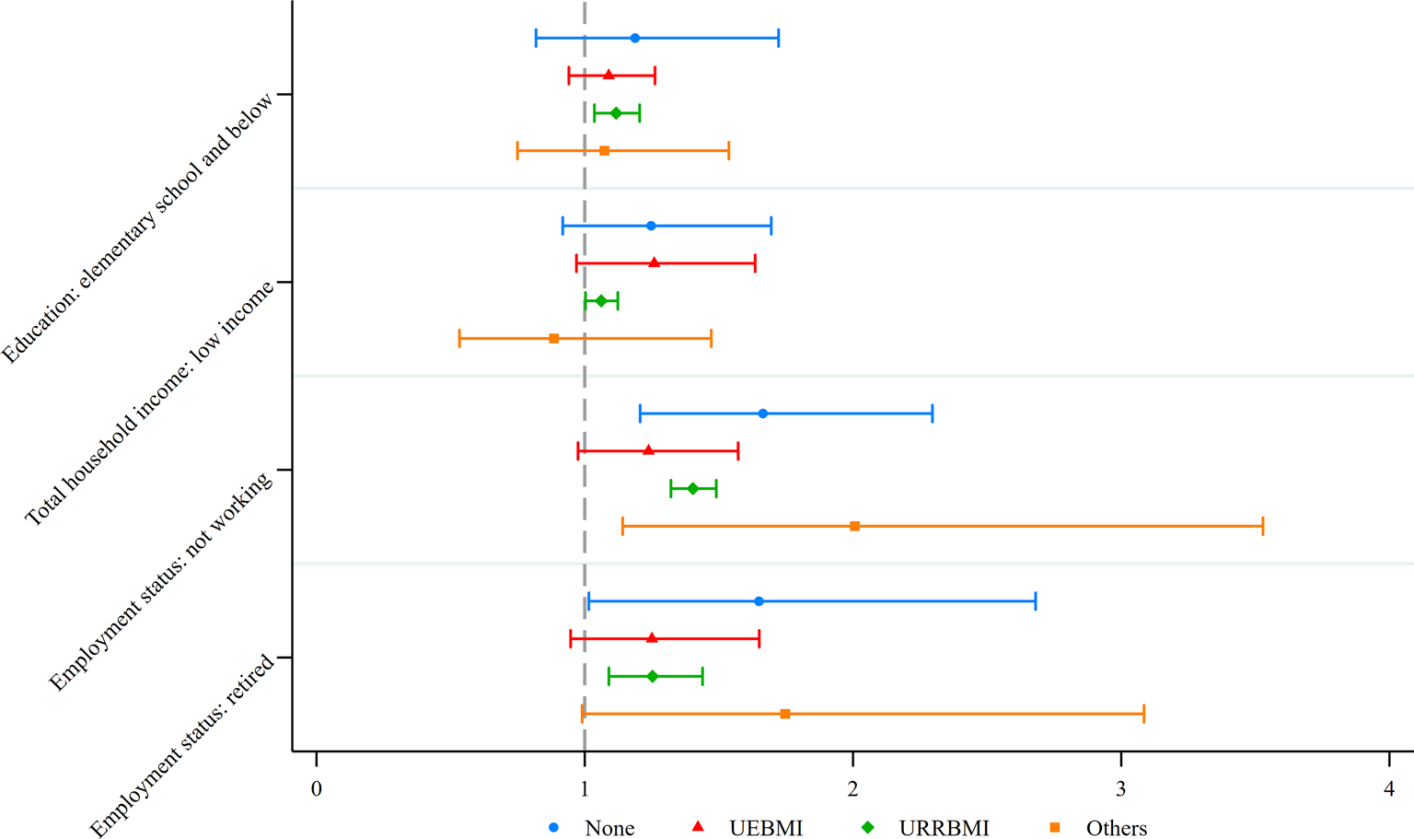
Association between socioeconomic status and inpatient utilization by health insurance. Notes: *UEBMI* Urban Employee Basic Medical Insurance; *URRBMI* Urban and Rural Resident Basic Medical Insurance. Others include government medical insurance, medical aid, private medical insurance, urban non-employed person’s health insurance, long-term care insurance, and other medical insurance. Models were adjusted for survey years, age, sex, residence, marital status, smoking, alcohol drinking, number of chronic diseases and self-reported health status

The results of the subgroup analysis based on health insurance coverage are presented in Fig. 4. Among participants enrolled in the UEBMI, there was no statistically significant association between SES and inpatient utilization (elementary school and below: IRR = 1.09, 95% CI: 0.94–1.26, *P* = 0.252; low income: IRR = 1.26, 95% CI: 0.97–1.64, *P* = 0.085; not working: IRR = 1.24, 95% CI: 0.97–1.57, *P* = 0.080, retired: IRR = 1.25, 95% CI: 0.95–1.65, *P* = 0.115). However, among those covered by the URRBMI, low SES was associated with higher inpatient utilization. Specifically, participants with elementary school and below (IRR = 1.12, 95% CI: 1.04–1.20, *P* = 0.004), low household income (IRR = 1.06, 95% CI: 1.00–1.23, *P* = 0.040), and those who were not working (IRR = 1.40, 95% CI: 1.32–1.49, *P* < 0.001) had higher inpatient utilization rates.

## Discussion 

This study undertook a comparative analysis of healthcare utilization trends among adults aged 50 or above in China and other countries and regions. The findings revealed that a significant upward trend in inpatient utilization rates in China over the past decade. By 2020, the inpatient utilization rates in CHARLS surpassed those observed in other international ageing cohorts. Furthermore, the study investigated the associations between SES and inpatient utilization in China. We found that low levels of education, household income, and being non-employed or retired were associated with higher rate of inpatient utilization. The associations between low SES and high inpatient utilization persisted among people enrolled in the URRBMI.

The upward trends in inpatient utilization rates observed in CHARLS stand in stark contrast to the declining trends in HRS and KLoSA. This trend reflects a dual driver: improved healthcare access for previously underserved populations and unintended overutilization. On one hand, the expansion of universal health insurance has effectively reduced financial barriers [11, 14], particularly for low-SES middle-aged and older adults who historically faced unmet inpatient needs. On the other hand, evidence suggests a non-negligible proportion of unnecessary hospitalizations. A study in large cities of eastern China found that 74.6% of inpatient admissions among adults aged over 60 years were avoidable, defined as conditions manageable via outpatient care [40]. If hospitalizations were purely need-based, we would expect a stronger correlation between multimorbidity severity and utilization—but our analysis showed that low-SES individuals with mild multimorbidity still had higher inpatient rates than high-SES individuals with moderate multimorbidity (Table S8). This mismatch is likely attributed to the distinctive structural characteristics of China’s hospital-centered healthcare systems, which are marked by underdeveloped primary care infrastructure. The weakness of community health services often results in patients bypassing primary care institutions and directly seeking treatment at tertiary hospitals, thereby generating induced demand for inpatient care. This structural imbalance has been further exacerbated by the continued expansion of hospital beds between 2011 and 2020 [41, 42], which may have inadvertently encouraged unnecessary hospitalizations.

In contrast, the gatekeeping systems widely adopted in some OECD countries have been effective in reducing unnecessary hospital admissions through stringent primary care referral mechanisms [43]. Gatekeeping means that patients have to see a primary care provider who decides whether specialist care is necessary. Such referral regulates the access to specialty care, hospital care, or diagnostic tests. It is supposed to give better control over the healthcare costs and more targeted and efficient hospital healthcare [44]. Continuity of care in general practice and access to a preferred general practitioner have been shown to reduce the emergency admission rates in general [45]. The institutional configuration of China’s healthcare systems appears particularly susceptible to hospitalization inflation, driven in part by its reimbursement policy framework. Notably, China’s current medical insurance reimbursement policy exhibits a pronounced financial bias toward inpatient services, creating economic incentives that may disproportionately drive hospitalization rates [46]. When combined with structural deficiencies in primary care delivery, this policy orientation presents unique challenges for healthcare resource allocation within the evolving landscape of China’s healthcare system.

Notably, existing international studies have primarily focused on specific populations (e.g., children, surgical patients, or those with specific diseases) [20–27], whereas our work adds evidence on SES disparities in a general ageing population, bridging a gap in cross-country comparisons of age-related healthcare utilization.

This study revealed higher inpatient utilization among middle-aged and older adults with low SES, a pattern consistent with findings from existing literature in the Netherlands. Individuals with low levels of education and household income used inpatient care more frequently than their counterparts with high education and income levels [16]. Individuals with lower educational attainment and income levels exhibited greater dependence on hospital services compared to those who were socioeconomically advantaged. The effect of low SES on frequent primary care attendance can be consistently seen in Germany [17]. Our findings differed from those in Brazil and Indonesia [18, 19], as we did not observe income- or education-based inequalities in inpatient utilization among middle-aged and older adults in China. This discrepancy likely stemmed from China’s health systems reform, which have aimed to expand access to healthcare through universal coverage and reduce financial barriers for vulnerable populations [46].

Additionally, our analysis showed that unemployed individuals had higher inpatient utilization compared to their employed counterparts, which is consistent with Danish findings [21, 23]. This may be related to work-related protective factors, such as employer-provided health benefits and healthier behaviors among workers. Work can have a positive effect on health when the working conditions are favorable and efforts remain below a moderate threshold [47]. These results underscore the complex interplay between socioeconomic determinants, health system policies, and utilization patterns in an ageing population.

This disparity in inpatient utilization among different SES populations likely originates from differences in health burdens, as our data indicate that low-SES groups are disproportionately affected by multimorbidity and report poorer self-rated health (Table S8). Health status can be perceived as proxy for the need for care and is a significant factor in explaining the relationship between SES and healthcare utilization. Lower-income and less-educated individuals were found to have poorer health conditions compared to their higher-income and higher-educated counterparts [18]. The unemployed have poorer health when compared to the employed population [48]. Due to relatively poorer healthcare access for the unemployed people, their mild symptoms or chronic exacerbations are more likely to progress to severe stages which require hospitalization, directly elevating inpatient needs. However, further research is needed to explore the mechanism of association between SES and inpatient utilization comprehensively.

The upward trends in inpatient utilization in China may also be intertwined with critical social dynamics, including urbanization and demographic shifts, which significantly shape healthcare access and utilization patterns. Rapid urbanization in China has led to uneven distribution of healthcare resources, with tertiary hospitals and high-quality medical services concentrated in urban areas [49]. This urban–rural divide may exacerbate the over-reliance on inpatient care among low-SES populations. Meanwhile, demographic factors, particularly the accelerating ageing process and changing population structure, further amplify these trends. The prevalence of chronic diseases among people aged 50 and above in China is as high as 80%, and the prevalence of comorbidities also increases with age [50]. Compared with high-SES counterparts, individuals with low SES experience an earlier onset of comorbidities and a faster accumulation of chronic conditions [51]. Unlike older adults with high SES who have access to home care and outpatient support, those with low SES lack such resources, which may lead to disease deterioration and subsequent hospitalization. China’s elderly population has grown rapidly, with a higher proportion of rural elderly facing both health vulnerabilities [52]. This demographic imbalance strengthens the association between low SES and high inpatient utilization.

We also found a socioeconomic gradient in inpatient utilization among China’s URRBMI population, with low-SES enrollees exhibiting significantly higher hospitalization rates. However, this association was not observed among the UEBMI enrollees. The UEBMI offers higher reimbursement rates for outpatient and inpatient visits and has a personal account for outpatient expenses. The more comprehensive coverage and higher reimbursement ratios under the UEBMI potentially attenuate SES-based disparities in healthcare access. The findings align with China’s broader trend of increased inpatient utilization following insurance expansions, as reflected in the 79.6% growth in inpatient care use between 2010 and 2018 [53]. Historical data confirm that pre-reform financial barriers substantially constrained healthcare access, particularly for vulnerable populations [46]. The introduction of health insurance programs has reduced the proportions of unmet needs for inpatient care, extended population coverage, and increased availability of healthcare services.

Our study had several limitations that should be considered when interpreting the results. First, the reliance on self-reported measures of healthcare utilization may have led to an underestimation of prevalence, particularly among older people and those of lower SES, who may be more inclined to under-report these factors [39]. This under-reporting could weaken the observed associations between low SES and higher utilization, as the true magnitude of disparities may be larger than our data capture. Second, the observational nature of the study precludes the establishment of causal associations between SES and healthcare utilization among adults aged 50 and above. Confounding variables that were not fully adjusted for—such as unmeasured health needs, healthcare-seeking preferences, or structural barriers to care—may drive both SES disparities and utilization patterns. Third, we lacked granular data on provider-side incentives, such as hospital bed occupancy rate targets that may drive unnecessary admissions. If healthcare providers prioritize meeting institutional targets, this could introduce supply-driven utilization that is unrelated to patient SES.

Despite the above limitations, our findings also have some implications for policy-making. First, this study underscores the need for systemic reforms to address the rising trend of inpatient utilization in China, which is driven by hospital-centric infrastructure, weak primary care, and policy incentives that favor inpatient services over outpatient visits. To mitigate unnecessary hospital admissions, strategies should focus on strengthening community health capacity. This can be achieved through workforce training and the implementation of gatekeeping systems similar to those in some OECD countries, which require primary referrals for tertiary care services. Second, tailored interventions are essential for vulnerable groups, especially those with low SES. Community-based chronic disease management programs can play a crucial role in improving health outcomes for these individuals. Additionally, health promotion initiatives that focus on early detection of chronic disease and encourage lifestyle modifications, coupled with financial incentives for adhering to primary care, could significantly reduce the incidence of avoidable hospitalizations among low-SES groups. Third, insurance integration efforts must prioritize equity, particularly given the persistent SES gradient among enrollees in the URRBMI program. There is a need to enhance outpatient coverage under this scheme to deter low-income groups from opting for hospitalization due to financial incentives. Simultaneously, expanding the risk-sharing model of the UEBMI with optimized actuarial designs could further reduce out-of-pocket burdens for vulnerable populations, thereby promoting more equitable access to healthcare services. Future research could further explore the specific drivers of healthcare utilization across populations with varying socioeconomic characteristics, with a focus on how interactions between policy incentives, healthcare resource allocation, and individual health behaviors shape choices between inpatient and outpatient services.

## Conclusions

This study compared healthcare utilization trends among adults aged 50 and above in China with other countries or regions, revealing a significant upward trend in China’s inpatient utilization rates over the past decade. By 2020, the inpatient utilization rates in CHARLS exceeded those of other ageing cohorts. Lower SES was associated with higher inpatient utilization, which may be attributed to the poorer health status of low-SES individuals. This association is still present in the URRBMI population. The study underscores the importance of systemic reforms to strengthen primary care infrastructure and align reimbursement policies with equitable access to healthcare services. Implementing targeted interventions for low-SES groups and accelerating health insurance integration are critical steps toward reducing avoidable hospitalizations and advancing healthcare equity, especially in the context of demographic shifts and an ageing population.

## Supplementary Information

Additional file1 (DOC 284 KB)

## Data Availability

The data that support the findings of this study are available to the public and available from the Gateway to Global Aging Data [https://g2aging.org/].

## Abbreviations

CHARLS: China Health and Retirement Longitudinal Study
CHCs: Community health centers
CI: Confidence intervals
HRS: Health and Retirement Study
IRR: Incidence rate ratio
KLoSA: Korean Longitudinal Study of Aging
SHARE: Survey of Health, Ageing and Retirement in Europe
OECD: Organization of Economic Co-operation and Development
SDGs: Sustainable development goals
SES: Socioeconomic status
UEBMI: Urban Employee Basic Medical Insurance
UHC: Universal health coverage
URRBMI: Urban and Rural Resident Basic Medical Insurance
