# Supplementary material for: Socioeconomic disparities in the trends of inpatient utilization for middle-aged and older adults in China: the perspective of international comparison from four cohorts
Source: Glob Health Res Policy. 2025 Dec 19;10:68. doi: 10.1186/s41256-025-00464-4 (PMC12715963; doi:10.1186/s41256-025-00464-4)
Supplement: Supplementary file 1 — Additional file1 (DOC 284 KB) [file 41256_2025_464_MOESM1_ESM.doc]

**Supplementary Information**

**Socioeconomic disparities in the trends of inpatient utilization for middle-aged and older adults in China: The perspective of international comparison from four cohorts**

Yemin Yuan, Huaxin Si, Yiqi Xia, Yanshang Wang, Zhenyu Shi, Ping He

**Contents:**

[Figure S1 Flowchart of sample selection in CHARLS, HRS, KLoSA, and SHARE 2](#__RefHeading___Toc32434)

[Table S1 Descriptive statistics in CHARLS, HRS, KLoSA, and SHARE 3](#__RefHeading___Toc25092)

[Figure S2 Flowchart of sample selection in CHARLS 4](#__RefHeading___Toc25371)

[Table S2 Description of health care utilization and in the four cohorts 5](#__RefHeading___Toc30498)

[Table S3 The annual number of outpatient visits and inpatient admissions per capita 6](#__RefHeading___Toc27604)

[Table S4 Sample Characteristics in CHARLS stratified by survey year 7](#__RefHeading___Toc22363)

[Table S5 The annual number of inpatient admissions per capita by socioeconomic status 8](#__RefHeading___Toc25632)

[Table S6 Association between socioeconomic status and inpatient utilization among middle-aged and older adults in China using Poisson models 9](#__RefHeading___Toc2872)

[Table S7 Cross-sectional analysis of socioeconomic status and inpatient utilization (n=44,137) 10](#__RefHeading___Toc15207)

[Table S8 Health behaviors and health status of different socioeconomic status (n=44,137) 11](#__RefHeading___Toc20832)

**Figure S1** Flowchart of sample selection in CHARLS, HRS, KLoSA, and SHARE

*CHARLS* China Health and Retirement Longitudinal Study; *HRS* Health and retirement study; *KLoSA* Korean Longitudinal Study of Aging; *SHARE* Survey of Health, Ageing and Retirement in Europe.

**Table S1 Descriptive statistics in CHARLS, HRS, KLoSA, and SHARE**

|  | CHARLS | HRS | KLoSA | SHARE |
| --- | --- | --- | --- | --- |
| Wave: date, n | wave 1: 2011, 6,186  wave 2: 2013, 7,990  wave 3: 2015, 6,227  wave 4: 2018, 10,624  wave 5: 2020, 13,704 | wave 11: 2012/13, 17,700  wave 12: 2014/15, 15,283  wave 13: 2016/18, 17,341  wave 14: 2018/19, 14,490  wave 15: 2020, 12,669 | wave 4: 2012, 7,476  wave 5: 2014, 7,006  wave 6: 2016, 7,468  wave 7: 2018, 6,920  wave 8: 2020, 6,446 | wave 4: 2011, 52,868  wave 5: 2013, 59,996  wave 6: 2015, 61,960  wave 7: 2017, 13,162  wave 8: 2019, 43,245 |
| Total number of respondents | 75,368 | 78,042 | 35,341 | 233,093 |
| Age (years), mean (SD) | 62.9 (9.0) | 67.5 (10.4) | 68.7 (10.1) | 67.7 (9.9) |
| Sex, n (%) |  |  |  |  |
| Male | 36,873 (48.9) | 33,527 (43.0) | 15,026 (42.5) | 102,722 (44.1) |
| Female | 38,495 (51.1) | 44,515 (57.0) | 20,315 (57.5) | 130,371 (55.9) |
| Residence, n (%) |  |  |  |  |
| Urban | 28,815 (38.2) | 56,111 (71.9) | 26,552 (75.1) | 155,673 (66.8) |
| Rural | 46,553 (61.8) | 21,931 (28.1) | 8,789 (24.9) | 77,420 (33.2) |
| Marital status, n (%) |  |  |  |  |
| Married or partnered | 63,469 (83.7) | 48,510 (62.2) | 26,698 (75.5) | 173,602 (74.5) |
| Unmarried and others^1^ | 11,899 (15.8) | 29,532 (37.8) | 8,643 (24.5) | 59,491 (25.5) |

*CHARLS* China Health and Retirement Longitudinal Study; *HRS* Health and retirement study; *KLoSA* Korean Longitudinal Study of Aging; *SHARE* Survey of Health, Ageing and Retirement in Europe; *SD* standard deviation.

^1^Others covered separated, divorced, and widowed.

**Figure S2** Flowchart of sample selection in CHARLS

*CHARLS* China Health and Retirement Longitudinal Study.

**Table S2 Description of** **health care utilization and in the** **four cohorts**

|  | **CHARLS** | **HRS** | **KLoSA** | **SHARE** |
| --- | --- | --- | --- | --- |
| Outpatient | Whether the respondent reports they have, in the past month, visited a public hospital, private hospital, public health center, clinic, or health worker’s or doctor’s practice, or been visited by a health worker or doctor for outpatient care. | Whether the respondent has visited a public health clinic or seen or talked to a medical doctor in the past two years. | | Whether the respondent reports any doctor visit in the last 12 months. For doctor visits, the question asks how many times the respondent has seen or talked to a medical doctor including emergency room or outpatient clinic visits. |
| Outpatient, number | The number of visits to any of the below medical facilities that the  respondents had during last month for outpatient treatment. | The number of times that the respondent has visited a public health clinic or has seen a doctor in the past two years. | | The reported number of doctor visits in the last 12 months. |
| Inpatient | Whether the respondent reports receiving any inpatient care in the past year. | Whether the respondent has been hospitalized at a hospital, nursing home, convalescent home or other long-term health care facility in the past two years. | | Whether the respondent reports any overnight hospital stay in the last 12 months. |
| Inpatient, number | The number of times that the respondent has received inpatient care in the past year. | The number of times that the respondent has been hospitalized at the hospital, nursing home, convalescent home or other long-term health care facility in the past two years. | | The reported number of hospital stays from 1 to 10 in the last 12 months. |

**Table S3 The annual number of outpatient visits and inpatient admissions per capita**

|  | Year | | | | | *tau-b* | *P* value |
| --- | --- | --- | --- | --- | --- | --- | --- |
|  | 2011-2012 | 2013-2014 | 2015-2016 | 2017-2018 | 2019-2020 |  |  |
| Outpatient visits per capita | | | | | | | |
| CHARLS | 5.10 | 6.33 | 4.97 | 4.16 | 5.67 | 0.00 | 0.269 |
| HRS | 4.90 | 4.72 | 4.86 | 4.57 | 4.25 | -0.02 | <0.001 |
| KLoSA | 5.79 | 5.93 | 6.36 | 6.42 | 6.26 | 0.02 | <0.001 |
| SHARE | 7.54 | 7.70 | 6.98 | 7.63 | 7.07 | 0.02 | <0.001 |
| Inpatient admissions per capita | | | | | | | |
| CHARLS | 0.14 | 0.22 | 0.23 | 0.29 | 0.36 | 0.08 | <0.001 |
| HRS | 0.20 | 0.19 | 0.20 | 0.19 | 0.16 | -0.02 | <0.001 |
| KLoSA | 0.08 | 0.07 | 0.06 | 0.06 | 0.05 | -0.05 | <0.001 |
| SHARE | 0.27 | 0.28 | 0.27 | 0.30 | 0.27 | 0.00 | 0.330 |

*CHARLS* China Health and Retirement Longitudinal Study; *HRS* Health and retirement study; *KLoSA* Korean Longitudinal Study of Aging; *SHARE* Survey of Health, Ageing and Retirement in Europe.

Table S4 Sample Characteristics in CHARLS stratified by survey year

| Characteristics | 2011  (n=6,180) | 2013  (n=7,914) | 2015  (n=6,138) | 2018  (n=10,622) | 2020  (n=13,283) |
| --- | --- | --- | --- | --- | --- |
| Socioeconomic status |  |  |  |  |  |
| Education, n (%) |  |  |  |  |  |
| Elementary school and below | 4,623 (74.8) | 5,781 (73.0) | 4,301 (70.1) | 6,834 (64.3) | 8,584 (64.6) |
| Middle school and above | 1,557 (25.2) | 2,133 (27.0) | 1,837 (29.9) | 3,788 (35.7) | 4,699 (35.4) |
| Total household income, n (%) |  |  |  |  |  |
| Low income | 3,169 (51.3) | 4,038 (51.0) | 3,175 (51.7) | 5,632 (53.0) | 6,628 (49.9) |
| High income | 3,011 (48.7) | 3,876 (49.0) | 2,963 (48.3) | 4,990 (47.0) | 6,655 (50.1) |
| Employment status, n (%) |  |  |  |  |  |
| Not working | 2,062 (33.4) | 2,318 (29.3) | 1,858 (30.3) | 2,815 (26.5) | 4,352 (32.8) |
| Working | 3,070 (49.7) | 4,208 (53.2) | 3,314 (54.0) | 6,031 (56.8) | 8,554 (64.4) |
| Retired | 1,048 (17.0) | 1,388 (17.5) | 966 (15.7) | 1,776 (16.7) | 377 ( 2.8) |
| Health insurance, n (%) |  |  |  |  |  |
| None | 395 ( 6.4) | 477 ( 6.0) | 353 ( 5.8) | 526 ( 5.0) | 178 ( 1.3) |
| UEBMI | 638 (10.3) | 786 ( 9.9) | 610 ( 9.9) | 1,213 (11.4) | 1,766 (13.3) |
| URRBMI | 4,935 (79.9) | 6,405 (80.9) | 4,988 (81.3) | 8,551 (80.5) | 11,193 (84.3) |
| Others^1^ | 212 ( 3.4) | 246 ( 3.1) | 187 ( 3.0) | 332 ( 3.1) | 146 ( 1.1) |
| Other covariates |  |  |  |  |  |
| Age (years), n (%) |  |  |  |  |  |
| 50-59 | 2,335 (37.8) | 2,817 (35.6) | 2,114 (34.4) | 3,976 (37.4) | 5,242 (39.5) |
| ≥60 | 3,845 (62.2) | 5,097 (64.4) | 4,024 (65.6) | 6,646 (62.6) | 8,041 (60.5) |
| Sex, n (%) |  |  |  |  |  |
| Male | 3,513 (56.8) | 3,840 (48.5) | 2,996 (48.8) | 5,191 (48.9) | 6,286 (47.3) |
| Female | 2,667 (43.2) | 4,074 (51.5) | 3,142 (51.2) | 5,431 (51.1) | 6,997 (52.7) |
| Residence, n (%) |  |  |  |  |  |
| Urban | 2,212 (35.8) | 2,786 (35.2) | 2,161 (35.2) | 4,242 (39.9) | 4,432 (33.4) |
| Rural | 3,968 (64.2) | 5,128 (64.8) | 3,977 (64.8) | 6,380 (60.1) | 8,851 (66.6) |
| Marital status, n (%) |  |  |  |  |  |
| Married or partnered | 1,083 (17.5) | 1,360 (17.2) | 1,098 (17.9) | 1,605 (15.1) | 2,032 (15.3) |
| Unmarried and others^2^ | 5,097 (82.5) | 6,554 (82.8) | 5,040 (82.1) | 9,017 (84.9) | 11,251 (84.7) |
| Smoking, n (%) | 2,975 (48.1) | 3,447 (43.6) | 2,815 (45.9) | 4,707 (44.3) | 5,501 (41.4) |
| Alcohol drinking, n (%) | 3,391 (54.9) | 3,497 (44.2) | 2,825 (46.0) | 5,088 (47.9) | 4,764 (35.9) |
| Number of chronic diseases, n (%) |  |  |  |  |  |
| 0 | 1,618 (26.2) | 1,896 (24.0) | 988 (16.1) | 1,815 (17.1) | 3,348 (25.2) |
| 1 | 1,821 (29.4) | 2,313 (29.2) | 1,477 (24.1) | 2,403 ( 22.6) | 3,061 (23.0) |
| ≥2 | 2,741 (44.4) | 3,705 (46.8) | 3,673 (59.8) | 6,404 (60.3) | 6,874 (51.8) |
| Self-reported health status, n (%) |  |  |  |  |  |
| Good | 1,458 (23.6) | 1,809 (22.9) | 1,334 (21.7) | 2,542 (23.9) | 9,819 (73.9) |
| Fair | 2,829 (45.8) | 4,173 (52.7) | 3,333 (54.3) | 5,186 (48.8) | 2,553 (19.2) |
| Poor | 1,893 (30.6) | 1,932 (24.4) | 1,471 (24.0) | 2,894 (27.2) | 911 ( 6.9) |

*CHARLS* China Health and Retirement Longitudinal Study; *UEBMI* Urban Employee Basic Medical Insurance; *URRBMI* Urban and Rural Resident Basic Medical Insurance. ^1^Others include government medical insurance, medical aid, private medical insurance, urban non-employed person’s health insurance, long-term care insurance, and other medical insurance. ^2^Others covered separated, divorced, and widowed.

**Table S5 The annual number of inpatient admissions per capita by socioeconomic status**

|  | Year | | | | | *tau-b* | *P* value |
| --- | --- | --- | --- | --- | --- | --- | --- |
|  | 2011 | 2013 | 2015 | 2018 | 2020 |
| Education |  |  |  |  |  |  |  |
| Elementary school and below | 0.17 | 0.24 | 0.25 | 0.30 | 0.35 | 0.07 | <0.001 |
| Middle school and above | 0.16 | 0.20 | 0.22 | 0.24 | 0.28 | 0.05 | <0.001 |
| *z* | -0.15 | 2.10 | 1.17 | 3.83 | 4.26 |  |  |
| *P* | 0.882 | 0.036 | 0.241 | <0.001 | <0.001 |  |  |
| Total household income |  |  |  |  |  |  |  |
| Low income | 0.16 | 0.24 | 0.23 | 0.31 | 0.36 | 0.08 | <0.001 |
| High income | 0.18 | 0.23 | 0.24 | 0.25 | 0.30 | 0.05 | <0.001 |
| *z* | -1.99 | 0.64 | -1.34 | 3.82 | 3.87 |  |  |
| *P* | 0.046 | 0.525 | 0.181 | <0.001 | <0.001 |  |  |
| Employment status |  |  |  |  |  |  |  |
| Not working | 0.22 | 0.32 | 0.33 | 0.41 | 0.46 | 0.11 | <0.001 |
| Working | 0.09 | 0.17 | 0.17 | 0.19 | 0.24 | 0.04 | 0.003 |
| Retired | 0.26 | 0.26 | 0.28 | 0.33 | 0.29 | 0.06 | <0.001 |
| *χ*^2^ | 79.81 | 67.92 | 80.24 | 197.31 | 168.11 |  |  |
| *P* | <0.001 | <0.001 | <0.001 | <0.001 | <0.001 |  |  |

**Table S6** Association between socioeconomic status and inpatient utilization among middle-aged and older adults in China using Poisson models

|  | Model 1 | |  | Model 2 | |  | Model 3 | |  | Model 4 | |
| --- | --- | --- | --- | --- | --- | --- | --- | --- | --- | --- | --- |
|  | IRR (95% CI) | *P* value |  | IRR (95% CI) | *P* value |  | IRR (95% CI) | *P* value |  | IRR (95% CI) | *P* value |
| Education (ref: middle school and above) | | | | | | | | | | | |
| Elementary school and below | 1.17 (1.10-1.25) | <0.001 |  | 1.24 (1.16-1.33) | <0.001 |  | 1.24 (1.15-1.33) | <0.001 |  | 1.16 (1.09-1.24) | 0.003 |
| Total household income (ref: high income) | | | | | | | | | | | |
| Low income | 1.14 (1.09-1.20) | <0.001 |  | 1.16 (1.10-1.21) | <0.001 |  | 1.15 (1.09-1.21) | <0.001 |  | 1.09 (1.04-1.15) | 0.007 |
| Employment status (ref: working) | | | | | | | | | | | |
| Not working | 1.86 (1.77-1.96) | <0.001 |  | 1.75 (1.66-1.85) | <0.001 |  | 1.73 (1.64-1.83) | <0.001 |  | 1.47 (1.40-1.55) | <0.001 |
| Retired | 1.53 (1.41-1.65) | <0.001 |  | 1.66 (1.52-1.81) | <0.001 |  | 1.65 (1.51-1.81) | <0.001 |  | 1.46 (1.34-1.60) | <0.001 |

*IRR* incidence rate ratio; *CI* confidence interval.

Model 1 was crude model. Model 2 was adjusted for survey years, age, sex, residence, marital status, and health insurance. based on Model 1. Model 3 was adjusted for smoking and alcohol drinking based on Model 2. Model 4 was adjusted for number of chronic diseases and self-reported health status based on Model 3.

**Table S7** Cross-sectional analysis of socioeconomic status and inpatient utilization (n=44,137)

|  | 2011 (n=6,180) | |  | 2013 (n=7,914) | |  | 2015 (n=6,138) | |  | 2018 (n=10,622) | |  | 2020 (n=13,283) | |
| --- | --- | --- | --- | --- | --- | --- | --- | --- | --- | --- | --- | --- | --- | --- |
|  | IRR (95% CI) | *P* value |  | IRR (95% CI) | *P* value |  | IRR (95% CI) | *P* value |  | IRR (95% CI) | *P* value |  | IRR (95% CI) | *P* value |
| Education (ref: middle school and above) | | | | | | | | | | | | | | |
| Elementary school and below | 1.11 (0.90-1.37) | 0.329 |  | 1.28 (1.08-1.51) | 0.004 |  | 1.04 (0.88-1.23) | 0.650 |  | 1.15 (1.02-1.29) | 0.027 |  | 1.12 (1.02-1.24) | 0.023 |
| Total household income (ref: high income) | | | | | | | | | | | | | | |
| Low income | 0.81 (0.67-0.98) | 0.028 |  | 1.05 (0.91-1.21) | 0.504 |  | 1.00 (0.87-1.15) | 0.971 |  | 1.18 (1.05-1.33) | 0.007 |  | 1.25 (1.14-1.37) | <0.001 |
| Employment status (ref: working) | | | | | | | | | | | | | | |
| Not working | 1.68 (1.38-2.04) | <0.001 |  | 1.49 (1.29-1.73) | <0.001 |  | 1.60 (1.38-1.87) | <0.001 |  | 1.47 (1.31-1.66) | <0.001 |  | 1.46 (1.33-1.61) | <0.001 |
| Retired | 2.19 (1.52-3.15) | <0.001 |  | 1.20 (0.92-1.57) | 0.180 |  | 1.36 (1.01-1.82) | 0.040 |  | 1.31 (1.09-1.59) | 0.005 |  | 1.19 (0.91-1.56) | 0.194 |

*IRR* incidence rate ratio; *CI* confidence interval.

Models were adjusted for: age, sex, residence, marital status, health insurance, smoking, alcohol drinking, number of chronic diseases and self-reported health status.

**Table S8 Health behaviors and health status of different socioeconomic status (n=44,137)**

|  | Education | | | |  | Total household income | | | |  | Employment status | | | | |
| --- | --- | --- | --- | --- | --- | --- | --- | --- | --- | --- | --- | --- | --- | --- | --- |
|  | Elementary school and below  (n=30,123) | Middle school and above  (n=14,014) | *χ*² | *P* value |  | Low income (n=22,642) | High income  (n=21,495) | *χ*² | *P* value |  | Not working (n=13,405) | Working (n=25,177) | Retired (n=5,555) | *χ*² | *P* value |
| Smoking, n (%) | |  | 610.86 | <0.001 |  |  |  | 18.99 | <0.001 |  |  |  |  | 310.23 | <0.001 |
| Yes | 12,071 (40.1) | 7,374 (52.6) |  |  |  | 9,748 (43.0) | 9,697 (45.1) |  |  |  | 5,063 (37.8) | 11,741 (46.6) | 2,641 (47.5) |  |  |
| No | 18,052 (59.9) | 6,640 (47.4) |  |  |  | 12,894 (57.0) | 11,798 (54.9) |  |  |  | 8,342 (62.2) | 13,436 (53.4) | 2,914 (52.5) |  |  |
| Alcohol drinking, n (%) | |  | 714.78 | <0.001 |  |  |  | 122.23 | <0.001 |  |  |  |  | 813.32 | <0.001 |
| Yes | 12,054 (40.0) | 7,511 (53.6) |  |  |  | 9,460 (41.8) | 10,105 (47.0) |  |  |  | 4,608 (34.4) | 12,040 (47.8) | 2,917 (52.5) |  |  |
| No | 18,069 (60.0) | 6,503 (46.4) |  |  |  | 13,182 (58.2) | 11,390 (53.0) |  |  |  | 8,797 (65.6) | 13,137 (52.2) | 2,638 (47.5) |  |  |
| Number of chronic diseases, n (%) | |  | 51.50 | <0.001 |  |  |  | 16.72 | <0.001 |  |  |  |  | 699.06 | <0.001 |
| 0 | 6,319 (21.0) | 3,346 (23.9) |  |  |  | 4,787 (21.2) | 4,878 (22.7) |  |  |  | 2,342 (17.5) | 6,342 (25.2) | 981 (17.7) |  |  |
| 1 | 7,562 (25.1) | 3,513 (25.1) |  |  |  | 5,689 (25.1) | 5,386 (25.1) |  |  |  | 3,009 (22.4) | 6,828 (27.1) | 1,238 (22.3) |  |  |
| ≥2 | 16,242 (53.9) | 7,155 (51.0) |  |  |  | 12,166 (53.7) | 11,231 (52.2) |  |  |  | 8,054 (60.1) | 12,007 (47.7) | 3,336 (60.0) |  |  |
| Self-reported health status, n (%) | |  | 663.07 | <0.001 |  |  |  | 636.35 | <0.001 |  |  |  |  | 1200.00 | <0.001 |
| Good | 10,613 (35.2) | 6,349 (45.3) |  |  |  | 7,789 (34.4) | 9,173 (42.7) |  |  |  | 4,592 (34.2) | 10,659 (42.3) | 1,711 (30.8) |  |  |
| Fair | 12,400 (41.2) | 5,674 (40.5) |  |  |  | 9,186 (40.5) | 8,888 (41.3) |  |  |  | 4,904 (36.6) | 10,230 (40.6) | 2,940 (52.9) |  |  |
| Poor | 7,110 (23.6) | 1,991 (14.2) |  |  |  | 5,667 (25.1) | 3,434 (16.0) |  |  |  | 3,909 (29.2) | 4,288 (17.1) | 904 (16.3) |  |  |
